# Perceived inadequate care and excessive overprotection during childhood are associated with greater risk of sleep disturbance in adulthood: the Hisayama Study

**DOI:** 10.1186/s12888-016-0926-2

**Published:** 2016-07-07

**Authors:** Mao Shibata, Toshiharu Ninomiya, Kozo Anno, Hiroshi Kawata, Rie Iwaki, Ryoko Sawamoto, Chiharu Kubo, Yutaka Kiyohara, Nobuyuki Sudo, Masako Hosoi

**Affiliations:** Department of Psychosomatic Medicine, Kyushu University Hospital, Fukuoka, 812-8582 Japan; Department of Epidemiology and Public Health, Graduate School of Medical Sciences, Kyushu University, Fukuoka, Japan; Division of Research Management, Center for Cohort Studies, Graduate School of Medical Sciences, Kyushu University, Fukuoka, Japan; Hisayama Research Institute for Lifestyle Diseases, Fukuoka, Japan; Department of Psychosomatic Medicine, Graduate School of Medical Sciences, Kyushu University, Fukuoka, Japan

**Keywords:** PBI, Parenting, Care, Overprotection, Sleep, PSQI

## Abstract

**Background:**

Sleep disturbance and poor sleep quality are major health problems worldwide. One potential risk factor for the development and maintenance of sleep disturbance is the parenting style experienced during childhood. However, its role in sleep disturbance in adulthood has not yet been estimated. This Japanese population study was done to clarify the relation between the parenting styles “care” and “overprotection” during childhood and sleep disturbance in adulthood.

**Methods:**

A total of 702 community-dwelling Japanese residents aged ≥ 40 years were assessed in 2011 for their perceptions of the parenting style of their parents by use of the Parental Bonding Instrument (PBI) and for sleep disturbance by use of the Pittsburgh Sleep Quality Index (PSQI). The odds ratio (OR) for sleep disturbance (a global PSQI score > 5) was calculated using a logistic regression model.

**Results:**

The prevalence of sleep disturbance was 29 %. After adjusting for sociodemographic, lifestyle, and physical factors in a comparison with the optimal parenting styles (high care and low overprotection), the ORs for sleep disturbance by men were significantly higher for low paternal care, by 2.49 times (95 % confidence interval [CI]: 1.21–5.09), and for high overprotection, by 2.40 times (95 % CI: 1.19–4.85), while the ORs were not significant for low maternal care and high overprotection. For women the only significant factor was high maternal overprotection, by 1.62 times (95 % CI: 1.05–2.52), while the ORs were not significant for low maternal care, low paternal care and high paternal overprotection. The association remained significant for high paternal overprotection for men after additionally controlling for depression.

**Conclusions:**

This study suggests that parenting style, especially inadequate care and excessive overprotection during childhood, is related to sleep disturbance in adulthood and that the association is much more significant for parents of the same sex as the child.

**Electronic supplementary material:**

The online version of this article (doi:10.1186/s12888-016-0926-2) contains supplementary material, which is available to authorized users.

## Background

Sleep disturbance and poor sleep quality are major health problems worldwide. Previous population-based studies have estimated the prevalence of insomnia and other sleep problems at from 10 % to 40 % [1]. Furthermore, insomnia and sleep disturbances are associated with increased healthcare costs due to high mortality, high comorbidity, reduced productivity, increased absenteeism, and accidents [2–7]. One potential factor that may effect sleep disturbance is the parenting style experienced during childhood [8]. A prospective study of infants demonstrated that hostile parenting predicts child sleep problems [9]. Infancy is a critical period, characterized by development of the sleep-wake cycle [10]. The parent’s behavior toward their baby, including waking-up, settling, napping, holding, feeding, and rocking, has been reported to have an effect on sleeping habits and the development of sleep disturbance confirmed by electroencephalogram [11–14]. Furthermore, infancy, childhood, and adolescence are critical periods for the development of hormonal reactions that resist stressors [15], which could influence sleep mechanisms.

Accumulating evidence indicates that perceived parental attitudes and behaviors, especially inadequate care and excessive overprotection as measured by the Parental Bonding Instrument (PBI) [16], are associated with an increased risk and severity of a wide variety of forms of disorders, such as suicide, mood disorder, obsessive-compulsive disorder, eating disorder, and inflammatory bowel disease [17–22]. Psychosomatic research has shown a relation between sleep problems and an insecure attachment style, one of the ways in which humans relate to others across their lifespan [23, 24]. Although an insecure attachment style has been correlated to an inadequate parenting style [25], there are no studies addressing the relation between the perceived parenting styles in childhood and sleep problems in adulthood. Furthermore, no studies have taken inadequate parenting style into consideration as a risk factor for sleep disturbance, although many factors are known that modulate sleep, including lifestyle, physical status, and psychological status [26, 27].

This Japanese population study, controlled for sociodemographic, lifestyle, physical, and psychological factors, was done to clarify the relation between parenting styles, particularly perceived inadequate care and overprotection during childhood, and sleep disturbance in adulthood,

## Methods

### Study participants

The data was gathered from participants in the Hisayama Study in 2011. The Hisayama Study is an ongoing, long-term, cohort study done to examine cardiovascular disease and its risk factors in Hisayama, a suburban town adjoining Fukuoka City, a metropolitan area in southwestern Japan, in which annual health checks and surveys have been done since 1961 [28]. The present study was conducted as a cross-sectional sub-study of the Hisayama Study for which participants were recruited in 2011. Of the 2,250 residents aged 40 years or older who participated in the 2011 survey, 860 (38.2 %) consented to participate in this study. After excluding 158 residents without data from the parenting questionnaire available for both parents, 702 (31.2 %, 265 men and 437 women) were enrolled for study.

### Assessment of sleep disturbance

Sleep disturbance was assessed by the Pittsburgh Sleep Quality Index (PSQI) [29], a self-reported questionnaire that assesses sleep disturbance and sleep quality during the past month that is widely used in clinical and population based settings [30–32]. It consists of 19 questions rated on a scale of 0–3 and is categorized into seven components: subjective sleep quality, sleep latency, sleep duration, habitual sleep efficiency, sleep disturbances, use of sleeping medications, and daytime dysfunction. The sum of the scores for these seven components yields a global score. We defined sleep disturbance as a global PSQI score greater than 5, which distinguishes patients from healthy controls with a high sensitivity and high specificity (89.6 % and 86.5 % of sleep disorder patients in the original version, 85.7 and 86.6 % of primary insomnia patients in the Japanese version) [29]. Internal reliability, test-retest reliability, and validity are acceptable in both versions [33].

### Assessment of parental bonding

Perceived parenting styles were measured using the Parental Bonding Instrument (PBI), a self-report questionnaire with 25 items that measures parenting styles in the first 16 years of life, as recalled by the respondents [16]. The PBI is scored separately for the father and mother to evaluate the relationship between the respondent and each parent, as they are subjectively perceived. The respondents are asked to score their parents’ attitudes and behaviors separately, using a 4-point Likert scale. Two subscales of parenting style are measured by the PBI: care and overprotection. The “care” subscale reflects perceived parental warmth, affection, and involvement contrasted with coldness and rejection. The “overprotection” subscale reflects perceived parental psychological over-control and intrusion contrasted with encouragement to psychological autonomy and exploration of the environment.

The parenting styles are divided into four categories (PBI quadrants) by dichotomized care and overprotection scores: “optimal bonding” (high care, low overprotection), “neglectful parenting” (low care, low overprotection), “affectionate constraint” (high care, high overprotection), and “affectionless control” (low care, high overprotection). “Affectionless control,” as proposed by Parker et al. [8] and confirmed by Sato et al. [34], is a maladaptive form of parenting that results in a particular vulnerability to the occurrence of psychopathology. The PBI score reflects the actual parenting attitude and is based on studies using corroborative witnesses and independent observers [35, 36]. The PBI has long-term stability [37] and its subscales have a high level of test-retest reliability and internal consistency [38]. The Japanese version of the PBI has been shown to have adequate validity [39].

### Measurement of potential confounding factors

A self-administered questionnaire concerning marital status, education, subjective economic status, occupation, current drinking, habitual smoking, habitual exercise, antihypertensive agent use, the use of insulin and oral glucose-lowering agents, existing pain, and past history of disease, including cardiovascular disease, cancer, respiratory disease and digestive disease, was completed by each participant and was checked by trained interviewers at the screening [28]. Body height and weight were measured in light clothing without shoes, and the body mass index (kg/m^2^) was calculated. Obesity was defined as a body mass index ≥ 25.0 kg/m^2^. Blood pressure was measured three times after the subject had rested for at least five minutes in the sitting position. The mean of the three measurements was used for the present analysis. Hypertension was defined as a systolic blood pressure ≥ 140 mmHg, diastolic blood pressure ≥ 90 mmHg, and/or current use of antihypertensive agents [40]. Blood glucose was measured by the glucose oxidase method. Diabetes mellitus was defined as a fasting plasma glucose level of ≥ 7.0 mmol/L (126 mg/dL), a 2-h post-loaded or causal glucose level of ≥ 11.1 mmol/L (200 mg/dL), HbA1c (NGSP) ≥ 6.5 %, and/or current use of insulin or oral glucose-lowering agents [41]. “Depressive symptom” as a psychological factor was measured using a self-report questionnaire, the Patient Health Questionnaire-9 (PHQ-9), which has been shown to be a reliable and valid assessment tool for both the diagnosis of depression and the evaluation of depression severity in primary care settings [42]. The PHQ-9 assesses the symptoms of depression over the past two weeks using nine questions rated on a 4-point scale; 0 (not at all) to 3 (nearly every day). The sum of the nine items is calculated to obtain a depression score ranging from 0 to 27. Higher scores indicate increased severity of depression. The validity of the Japanese version of the PHQ-9 has been confirmed [43].

### Statistical analysis

Comparisons of the care and overprotection scores of men and women were performed by Mann-Whitney *U*-test. We dichotomized care and protection scores according to median scores: low score for care < 28 for fathers and < 31 for mothers and high score for overprotection ≥ 8 for both fathers and mothers. To account for the interaction between the care and overprotection subscales, paternal and maternal bonding was classified into four quadrants: “high care, low overprotection” (optimal bonding), “low care, low overprotection” (neglectful parenting), “high care, high overprotection” (affectionate constraint), and “low care, high overprotection” (affectionless control). Comparisons of characteristics between the high and low parenting subscales (care and overprotection) were performed by a Student *t*-test for parametric continuous variables, a Mann-Whitney *U* test for non-parametric ordinal variables, or a Chi-square test for dichotomous variables. Odds ratios (ORs) and the confidence interval (CI) of the parenting styles on the presence of sleep disturbance were estimated by logistic regression analysis, with adjustment for sociodemographic and lifestyle factors (Model 1: age, marital status, educational level, subjective economic level, occupation, current drinking, current smoking, and habitual exercise), physical factors (Model 2: model 1 + obesity, hypertension, diabetes, past history of cardiovascular disease, past history of cancer, past history of respiratory disease, past history of digestive disease, and pain symptom) and a psychological factor (Model 3: model 2 + depressive symptom score). In addition, we performed sensitivity analyses that used the quartiles of the parenting scores in logistic regression analysis. The heterogeneity in the association of age (40–64 as middle age and 65–96 as old age) was assessed by adding the interaction term to the relevant logistic model.

The SAS software package version 9.2 (SAS Institute, Cary, NC, USA) was used for all analyses. Two-sided values of *p* < 0.05 were considered significant.

## Results

The parental care and overprotection scores for all participants are summarized by gender in Table 1. The parental care scores were significantly higher and overprotection scores were significantly lower for women than for men.

**Table 1:** Gender based parental care and overprotection scores for all participants

Parenting factors		Score range	All (*n* = 702)		Men (*n* = 265)		Women (*n* = 437)		*p* value^a)^
			median	IQR	median	IQR	median	IQR	
Father									
	Care	0–36	28	21–33	26	20–32	30	23–34	<0.001
	Overprotection	0–39	8	4–13	9	4–14	8	3–13	0.03
Mother									
	Care	0–36	31	25–35	29	24–34	32	26–35	<0.001
	Overprotection	0–39	8	4–13	9	4–14	7	3–13	0.02

IQR, interquartile range

^a)^P values between men and women were tested by Mann-Whitney U-test.

The characteristics of all participants, classified by the parental care and overprotection scores of their father and mother, are summarized in Table 2. The frequency of men, low subjective economic level, current pain, and the median score for depressive symptom were significantly higher for participants who experienced than did not experience an inadequate parenting style (low care or high over-protection), regardless of the sex of the parent. Additionally, participants with low paternal care had significantly higher frequencies of current smoking and drinking than those without, whereas significant increases in the frequencies of diabetes and past history of cardiovascular disease were observed among subjects with high paternal over-protection. The frequency of a past history of digestive disease was significantly increased for participants with either low paternal care or high paternal over-protection as compared with those without. For the maternal parenting styles, low maternal care was significantly associated with higher frequencies of low educational level and diabetes. Participants with high maternal over-protection were likely to be younger and had significantly higher frequencies of current smoking and a past history of cancer than did those without. Additional information, the characteristics for all participants according to sleep disturbance are shown in Additional file 1: Table S1.

**Table 2:** Characteristics for all participants according to parental care and overprotection

	Father				Mother			
	High care	Low care	Low over-protection	High over-protection	High care	Low care	Low over-protection	High over-protection
	(*n* = 363)	(*n* = 339)	(*n* = 327)	(*n* = 375)	(*n* = 358)	(*n* = 344)	(*n* = 337)	(*n* = 365)
Sociodemographic and life style factors								
Age, mean (SD)	59.7 (10.9)	58.9 (11.2)	59.1 (10.7)	59.4 (11.4)	59.4 (11.2)	59.1 (10.9)	60.3 (10.4)	58.4 (11.5)^*^
Sex, male (%)	28.1	48.1^**^	33.3	41.6^*^	30.7	45.1^**^	32.6	42.4^**^
Marital status, without partner (%)	20.1	18.6	18.7	20.0	17.3	21.5	19.3	19.5
Educational level, under 10 years (%)	11.8	15.0	11.6	14.9	10.6	16.3^*^	12.5	14.3
Subjective economic level, low-very low (%)	15.2	26.6^**^	14.7	25.9^**^	13.7	27.9^**^	16.0	24.9^**^
Occupation, unemployed (%)	50.7	49.0	47.4	52.0	49.4	50.3	49.9	49.9
Current smoking, yes (%)	8.0	13.9^*^	8.9	12.5	9.2	12.5	7.7	13.7^*^
Current drinking, yes (%)	50.4	58.7^*^	53.8	54.9	53.4	55.5	53.1	55.6
Habitual exercise, yes (%)	53.2	52.5	53.8	52.0	53.1	52.6	54.9	51.0
Physical factors								
Obesity, BMI >25 (%)	22.0	26.0	25.1	23.0	22.4	25.6	23.4	24.4
Hypertension (%)	41.3	42.2	39.5	43.7	40.8	42.7	41.5	41.9
Diabetes (%)	13.0	17.1	11.0	18.4^**^	12.0	18.0^*^	12.5	17.3
Past history of cardiovascular diseases (%)	11.3	15.9	10.7	16.0^*^	11.5	15.7	11.3	15.6
Past history of cancer (%)	5.8	6.5	4.6	7.5	5.9	6.4	3.9	8.2^*^
Past history of respiratory diseases (%)	13.0	16.5	12.5	16.5	13.4	16.0	12.8	16.4
Past history of digestive diseases (%)	16.5	25.4^**^	15.3	25.6^**^	17.9	23.8	19.3	22.2
Current pain symptom (%)	55.9	67.0^**^	54.7	66.9^**^	57.5	65.1^*^	56.4	65.8^*^
Psychological factor								
Depression symptom, Score, median (IQR)	2 (0–4)	3 (1–6)^**^	2 (0–4)	3 (1–6)^**^	2 (0–3)	3 (1–6)^*^	2 (0–4)	3 (1–6)^*^

Hypertension was defined as blood pressure ≥ 140/90 mm Hg and/or use of an antihypertensive agent. Diabetes was defined as a fasting plasma glucose level of ≥ 7.0 mmol/L (126 mg/dL), and/or a 2-h post-loaded or causal glucose level of ≥ 11.1 mmol/L (200 mg/dL), HbA1c (NGSP) ≥ 6.5 % and/or current use of insulin or oral glucose-lowering agents

Hypertension was defined as blood pressure ≥ 140/90 mm Hg and/or use of an antihypertensive agent. Diabetes was defined as a fasting plasma glucose level of ≥ 7.0 mmol/L (126 mg/dL), and/or a 2-h post-loaded or causal glucose level of ≥ 11.1 mmol/L (200 mg/dL), HbA1c (NGSP) ≥ 6.5 % and/or current use of insulin or oral glucose-lowering agents

Values were tested by *t*-test (for age), Chi-square test (for frequencies) or Mann-Whitney *U*-test (for depression)

* *p* < 0.05, ** *p* < 0.01 Values are expressed as mean (standard deviation [SD]), frequency or median

Table 3 shows the odds ratios for sleep disturbance according to parenting style, classified by sex. The overall prevalence of sleep disturbance was 29 % (203/702). The ORs for sleep disturbance were significantly higher for men with low paternal care and high overprotection and for both sexes for high maternal overprotection than were found for an adequate parenting style after adjustment for sociodemographic and lifestyle factors (Model 1). After adjustment for the covariates in Model 1 plus physical factors (Model 2), the associations were attenuated, but the association with low paternal care and high overprotection for men and with high maternal overprotection for women remained significant. Moreover, after further adjustment for depressive symptom as a psychological factor (Model 3), the significant association remained only for high paternal overprotection for men. The findings were not substantially altered when sensitivity analyses were done using the quartile categories of the parenting scores (Additional file 3: Table S3). We also assessed the heterogeneity in the magnitude of these associations for age-based subgroups (40–64 as middle age and 65–96 as old age), resulting in no observed evidence of significant heterogeneity (all *p* for heterogeneity > .094).

**Table 3:** Odds ratios for sleep disturbance according to parenting styles, by sex

Inadequate parenting styles	(vs. adequate parenting style)	No. with sleep disturbance		Model 1 (Adjusted for socio- demographic and life style factors)		Model 2 (Model 1+ physical factors)		Model 3 (Model 2+ psychological factor)	
		Inadequate parenting	Adequate parenting	OR (95 % CI)	*p* value	OR (95 % CI)	*p* value	OR (95 % CI)	*p* value
Men (*n* = 265)									
Father									
Low care	(vs. High care)	51/163	15/102	2.67 (1.36–5.20)	0.004	2.49 (1.21–5.09)	0.01	1.79 (0.82–3.87)	0.1
High overprotection	(vs. Low overprotection)	50/156	16/109	2.91 (1.50–5.65)	0.002	2.40 (1.19–4.85)	0.01	2.17 (1.03–4.58)	0.04
Mother									
Low care	(vs. High care)	43/155	23/110	1.50 (0.81–2.79)	0.2	1.47 (0.76–2.85)	0.2	1.18 (0.58–2.40)	0.6
High overprotection	(vs. Low overprotection)	46/155	20/110	2.10 (1.12–3.97)	0.02	1.87 (0.95–3.65)	0.07	1.59 (0.77–3.28)	0.2
Women (*n* = 437)									
Father									
Low care	(vs. High care)	57/176	80/361	1.04 (0.67–1.61)	0.8	0.95 (0.60–1.49)	0.8	0.67 (0.41–1.09)	0.1
High overprotection	(vs. Low overprotection)	75/219	62/218	1.23 (0.81–1.88)	0.3	1.13 (0.73–1.75)	0.6	0.91 (0.57–1.45)	0.7
Mother									
Low care	(vs. High care)	70/189	67/248	1.48 (0.96–2.25)	0.07	1.37 (0.89–2.13)	0.2	0.98 (0.61–1.58)	0.9
High overprotection	(vs. Low overprotection)	77/210	60/227	1.68 (1.10–2.56)	0.02	1.62 (1.05–2.52)	0.03	1.28 (0.80–2.04)	0.3

Model 1: Adjusted for socio-demographic and life style factors (age, marital status, educational level, subjective economic level, occupation, current drinking, current smoking, and habitual exercise)

Model 2: Adjusted for covariates included in Model 1 + physical factors (Obesity, hypertension, diabetes, past history of cardiovascular disease, past history of cancer, past history of respiratory disease, past history of digestive disease, and current pain symptom.)

Model 3: Adjusted for covariates included in Model 2 + psychological factor (depressive symptom)

To examine the combined effects of care and overprotection, we calculated ORs for sleep disturbance according to combinations of the parenting styles of the parent of the same sex (i.e. father-son and mother-daughter) because the sample size was limited and the parenting style of the parent of the same sex was likely to be related to sleep disturbance (Table 4). The ORs for sleep disturbance were significantly higher for high care - high overprotection (affectionate constraint) and low care - high overprotection (affectionless control) than for high care - low overprotection (optimal bonding) after adjustment for sociodemographic and lifestyle factors and further adjustment for physical factors in model 2. Additionally, the influence of low care - high overprotection (affectionless control) on sleep disturbance became non-significant after further adjustment for depressive symptom, whereas the association of high care - high overprotection (affectionate constraint) remained significant (model 3).

**Table 4:** ORs for sleep disturbance by combinations of the parenting styles by the same sex parent

Parenting style ^a^	No. of participants	No. with sleep disturbance	Model 1		Model 2		Model 3	
			OR (95 % CI)	*p* value	OR (95 % CI)	*p* value	OR (95 % CI)	*p* value
HC-LO	250	51	1.00		1.00		1.00	
LC-LO	86	25	1.66 (0.93–2.95)	0.08	1.62 (0.90–2.91)	0.1	1.28 (0.69–2.39)	0.4
HC-HO	100	31	1.90 (1.11–3.26)	0.02	1.85 (1.07–3.22)	0.03	1.83 (1.03–3.26)	0.04
LC-HO	266	96	2.24 (1.48–3.41)	<0.001	1.98 (1.29–3.05)	0.002	1.42 (0.89–2.27)	0.1

*HC-LO* High Care-Low overprotection, *LC-LO* Low Care-Low Overprotection, *HC-HO* High Care-High overprotection, *LC-HO* Low Care-High Overprotection

^a^Men categorized by the father’s parenting styles and women by the mother’s parenting styles

Model 1: Adjusted for sociodemographic and lifestyle factors (age, sex, marital status, educational level, subjective economic level, occupation, current drinking, current smoking, and habitual exercise.)

Model 2: Adjusted for Model 1 + physical factors (Obesity, hypertension, diabetes, past history of cardiovascular disease, past history of cancer, past history of respiratory disease, past history of digestive disease, and current pain symptom.)

Model 3: Adjusted for Model 2 + depressive symptom as a psychological factor

## Discussion

To our knowledge, this is the first population study to examine the associations between perceived parenting styles during childhood and sleep disturbance in adulthood. The perception of both parenting styles and sleep disturbance is important in the clinical setting, even though the current perception of the parenting style may differ from what may have actually been experienced. Therefore, we focused on the relation between the perceived parenting styles and sleep disturbance in the self-reported questionnaires of this study. After adjusting for sociodemographic, lifestyle, and physical factors, low paternal care and high overprotection were associated with the sleep disturbance of men, as was high maternal overprotection for women. Additionally, further adjustment for depressive symptom attenuated these associations, with the result that only high paternal overprotection remained significant for men. The findings of this study suggest that parenting style, especially inadequate care and excessive overprotection during childhood, is related to sleep disturbance in adulthood and that the association is more significant for same sex parent-offspring pairs. Furthermore, these associations may be partially mediated by physical factors and depression.

### Comparison with previous reports

As far as we know there are no studies of the relation between parenting style and the sleep disturbance of adults, but several studies have addressed the relationship between insecure attachment, which is known to be closely related to inadequate parenting (i.e. low care and high overprotection) [25], and sleep disturbance. These previous studies, in accord with ours, showed that insecure attachment is a risk factor for sleep disturbance and lower quality of sleep across the lifespan [23], but they did not directly evaluate parenting styles.

### Effects of the parenting style of the parent of the same sex

The present study demonstrated that the parenting style of the parent of the same sex (i.e. father for men and mother for women) is more likely to be related to sleep disturbance. Ohtani et al. demonstrated that healthy Japanese volunteers who experienced inadequate parenting from the parent of the same sex have higher interpersonal sensitivity scores, from an analysis of covariance test, than do those who experienced adequate parenting, whereas no such association was observed for inadequate parenting by the parent of the opposite sex. [44, 45]. Interpersonal sensitivity, which is affected by inadequate same sex parenting, may be related to sleep disturbance through a personality factor that induces interpersonal stress.

### Gender differences in the relation of parenting to sleep disturbance

This study found different influences of the parenting style of parents of the same sex on sleep disturbance. For men, low paternal care was significantly associated with a higher likelihood of sleep disturbance, but this significant association was diminished after adjusting for depressive symptom. Previous studies have reported that low paternal care is associated with the treatment-resistant depression of outpatients with major depression and with the depression of male adolescent delinquents [46, 47]. These findings raise the possibility that the sleep disturbance of men with low paternal care can be mainly attributed to depression. In contrast, there was no evidence of a relation between low maternal care and the sleep disturbance of women.

Maternal overprotection was significantly associated with a higher likelihood of sleep disturbance for women, after adjustment for sociodemographic, lifestyle and physical factors, but further adjustment for depressive symptom attenuated this association. Previous studies suggested that maternal overprotection is related to psychological factors such as depression [20, 48], supporting the hypothesis that maternal overprotection is related to the sleep disturbance of women via depressive symptoms. By contrast, the association between high paternal overprotection and the sleep disturbance of men was significant even after adjustment for depressive symptom. Sadeh et al. reported that infants who fall asleep with significant parental involvement (i.e., while being held, fed, rocked, etc.) are more likely to have an increased number and duration of night waking than infants who fall asleep in their crib with minimal parental assistance. It is assumed that infants who fall asleep with excessive parental overprotection fail to develop their own self-regulation and soothing skills and, therefore, continue to rely on repeated parental intervention during the night. These cognitions and behaviors may be related to sleep habits across the lifespan and contribute to sleep disturbance in adulthood. Men with high paternal overprotection in childhood may have mechanisms other than depression that contribute to sleep disturbance.

The precise reason for the gender related discrepancy in the influence of parenting styles is unclear, but it is possible that men are more susceptible than women to the parenting style during childhood [49]. Further investigations will be necessary to clarify the reasons for the differences.

### The relation of combinations of parenting styles to sleep disturbance

In this study, high care with high overprotection (affectionate constraint) was associated with sleep disturbance after controlling for physical health factors and depressive symptom, despite previous findings that high care is a protective factor for psychological and physiological diseases. Parker and Lipscombe have shown that the combination of high care and high overprotection induces hypochondria and a tendency to be dependent [50]. Therefore, people who have experienced high care and high overprotection may be likely to be overly sensitive and to complain of sleep disturbances. Further research will be needed to examine sleep disturbance that uses both subjective and objective measures (i.e. polysomnography) to determine the extent to which this association is due to reported or objective differences.

The present study demonstrated that the parenting style combination of low care - high overprotection (affectionless control) in childhood increases the risk of sleep disturbance in adulthood. In European studies of the epidemiology of mental disorders, the authors reported that the parenting style “affectionless control” was associated with anxiety disorder, a risk factor for sleep disturbance that could mediate the association [51, 52]. Selterman and Drigotas have reported that students with insecure attachment, which is known to be closely affected by parenting style (low care and high overprotection), experienced significantly more stress and conflict in their dreams compared with students with secure attachment [53]. Therefore, stressful dreams may contribute to the sleep disturbance of people who experienced low care - high overprotection parenting. The significant association between affectionless control and sleep disturbance disappeared after additionally controlling for physical factors and depressive symptom. Previous studies have reported that low care - high overprotection is associated with physical symptoms (i.e. chronic pain, respiratory disease, and inflammatory bowel disease) and depression [17, 20, 22, 48, 54]. These physical and depressive symptoms may mediate the association.

### Limitations

This study has several limitations. First, the assessment of parenting styles was based on a self-reported, retrospective measure. The PBI is a stable questionnaire that has been shown to have adequate test-retest reliability for a retrospective period of 20 years [37]. Because the PBI score reflects the actual parenting attitude, based on studies using corroborative witnesses and independent observers, it would seem to have an acceptable level of objectivity. However, we cannot rule out the possibility that current mood influenced the subjective appraisal of recalled events about parenting [55]. Additionally, if the participant could not recall environmental information in childhood, Osypuk et al. suggested that associations with health may be biased towards the null [56]. Second, we used only a self-reported questionnaire for measuring sleep disturbance. Although the PSQI questionnaire is widely used for assessing subjective complaints and sleep quality, future studies should add objective measures such as actigraphy. Third, the data are cross-sectional. We thus cannot deny the possibility of reverse causality; that sleep disturbance influenced the rating of the parenting styles. Prospective longitudinal studies are needed to clarify the contribution of inadequate parenting style to the development of sleep disturbance. Fourth, there is the possibility of selection bias because approximately two-thirds of the individuals who participated in the regular Hisayama Study survey did not participate in our research. In an analysis comparing the characteristics of subjects included and excluded, relatively healthy people were suggested to have been more likely to participate in the study than non-healthy people: subjects included in this study were younger and there was a lower frequency of current smokers, hypertensive patients, and a clinical past history of cardiovascular disease, cancer, and digestive disease than subjects excluded (Additional file 2: Table S2). Therefore, the generalizability of our findings to all individuals in the community may be limited. Nevertheless, we believe that our findings provide important information on the influence of inadequate parenting style on sleep disturbance. Finally, the parenting styles could be different by race and culture. Therefore, care must be taken when generalizing our results of the relation between parenting style and sleep disturbance to other countries and cultures, even though they are consistent with those of previous studies about the negative effect of low care and high overprotection on physical or psychological diseases done in other countries [17, 20, 48].

## Conclusions

The findings of this population study suggest that inadequate care and excessive overprotection during childhood are related to sleep disturbance in adulthood and that the association is much more significant when associated with the parent of the same sex. Furthermore, these associations were likely to be mediated by physical health factors and depression. Mass-education and social support for optimal parenting that generates a secure attachment would be a promising initiative for the prevention of sleep disturbance, which would be of great social benefit from the viewpoints of global health and reducing the economic burden of both patients and medical systems. Further prospective and interventional studies will be needed to clarify the mechanisms responsible for the relation of parenting style to sleep disturbance.

## Abbreviations

BMI, body mass index; CI, confidence interval; IQR, interquartile range; ORs, odds ratios; PBI, Parental Bonding Instrument; PHQ-9, Patient Health Questionnaire-9; PSQI, Pittsburgh Sleep Quality Index; SD, standard deviation

## Additional files

Additional file 1: Table S1. Characteristics for all participants according to sleep disturbance. (DOCX 18 kb) Additional file 2: Table S2. Odds ratios for sleep disturbance according to parental bonding style. (DOCX 30 kb) Additional file 3: Table S3. Characteristics of participants and non-participants. (DOCX 15 kb)
