# Supplementary material for: Perceived inadequate care and excessive overprotection during childhood are associated with greater risk of sleep disturbance in adulthood: the Hisayama Study
Source: BMC Psychiatry. 2016 Jul 7;16:215. doi: 10.1186/s12888-016-0926-2 (PMC4936292; doi:10.1186/s12888-016-0926-2)
Supplement: Additional file 1: Table S1. — Characteristics for all participants according to sleep disturbance. (DOCX 18 kb) [file 12888_2016_926_MOESM1_ESM.docx]

| **Table S1. Characteristics for all participants according to sleep disturbance.** | | | | | |
| --- | --- | --- | --- | --- | --- |
|  |  | All study sample | Sleep disturbance (-) | Sleep disturbance (+) | *p* value |
|  |  | (n=702) | (n=499) | (n=203) |  |
| **Sociodemographic and life style factors** | | |  |  |  |
|  | Age, mean±SD | 59.3±11.0 | 58.7±11.0 | 60.7±11.1 | 0.03 |
|  | Sex, male (%) | 37.8 | 39.9 | 32.5 | 0.07 |
|  | Marital status, without partner (%) | 19.4 | 17.6 | 23.7 | 0.07 |
|  | Educational level, under 10 years (%) | 13.4 | 12.8 | 14.8 | 0.5 |
|  | Subjective economic level, low-very low (%) | 20.7 | 17.4 | 28.6 | 0.001 |
|  | Occupation, unemployed (%) | 49.9 | 47.3 | 56.2 | 0.03 |
|  | Current smoking, yes (%) | 10.8 | 11.2 | 9.9 | 0.7 |
|  | Current drinking, yes (%) | 54.4 | 56.1 | 50.3 | 0.2 |
|  | Habitual exercise, yes (%) | 52.8 | 54.7 | 48.3 | 0.1 |
|  |  |  |  |  |  |
| **Physical factors** | |  |  |  |  |
|  | Obesity, BMI ≥ 25 (%) | 23.9 | 24.1 | 23.7 | 0.9 |
|  | Hypertension (%) | 41.7 | 40.5 | 44.8 | 0.3 |
|  | Diabetes (%) | 15.0 | 14.0 | 17.2 | 0.3 |
|  | Past history of cardiovascular diseases (%) | 13.5 | 13.2 | 14.3 | 0.7 |
|  | Past history of cancer (%) | 6.1 | 5.4 | 7.9 | 0.2 |
|  | Past history of respiratory diseases (%) | 14.7 | 11.6 | 22.2 | <0.001 |
|  | Past history of digestive diseases (%) | 20.8 | 19.4 | 24.1 | 0.2 |
|  | Current pain symptom (%) | 61.3 | 56.5 | 72.9 | <0.001 |
|  |  |  |  |  |  |
| **Psychological factor** | |  |  |  |  |
|  | Depression symptom, score, median | 2 (1-5) | 2 (0-4) | 4 (2-7) | <0.001 |
| Values are expressed as mean ± S.D., median (interquartile range) or frequency. | | | | | |
| Hypertension was defined as blood pressure ≥140/90 mm Hg and/or use of an antihypertensive agent. | | | | | |
| Diabetes was defined as a fasting plasma glucose level ≥ 7.0 mmol/L (126 mg/dL), and/or 2-h post-loaded or causal glucose level ≥ 11.1 mmol/L (200 mg/dL), HbA1c (NGSP) ≥ 6.5% and/or current use of insulin or oral glucose-lowering agents. | | | | | |
| Values were tested by t-test (for age), Mann-Whitney U test (for depression) or Chi-square test (for frequencies). | | | | | |
