# Supplementary material for: Perceived inadequate care and excessive overprotection during childhood are associated with greater risk of sleep disturbance in adulthood: the Hisayama Study
Source: BMC Psychiatry. 2016 Jul 7;16:215. doi: 10.1186/s12888-016-0926-2 (PMC4936292; doi:10.1186/s12888-016-0926-2)
Supplement: Additional file 2: Table S2. — Odds ratios for sleep disturbance according to parental bonding style. (DOCX 30 kb) [file 12888_2016_926_MOESM2_ESM.docx]

| **Table S2. Odds ratios for sleep disturbance according to parental bonding style.** | | | | | | | | |  |  |  |  |  |  |  |
| --- | --- | --- | --- | --- | --- | --- | --- | --- | --- | --- | --- | --- | --- | --- | --- |
| Quartiles of parenting scores | No. of subjects | No. with sleep disturbance |  | Model 1 |  |  |  | Model 2 | |  |  |  | Model 3 |  |  |
|  |  |  |  | Sociodemographic and lifestyle factors adjusted | | |  | Physical factors adjusted | | | |  | Psychological factor adjusted | | |
|  |  |  |  | OR (95%CI) | *P* value | *P* for trend |  | OR (95%CI) | | *P* value | *P* for trend |  | OR (95%CI) | *P* value | *P* for trend |
| ***Men (n=265)*** |  |  |  |  |  |  |  |  | |  |  |  |  |  |  |
| Father |  |  |  |  |  |  |  |  | |  |  |  |  |  |  |
| Care |  |  |  |  |  |  |  |  | |  |  |  |  |  |  |
| Q4 (≥32) | 69 | 11 |  | 1.00 |  | 0.004 |  | 1.00 | |  | 0.02 |  | 1.00 |  | 0.3 |
| Q3 (26-31) | 64 | 12 |  | 1.01 (0.39-2.61) | 1.0 |  |  | 1.00 (0.37-2.69) | | 1.0 |  |  | 0.96 (0.34-2.69) | 0.9 |  |
| Q2 (21-25) | 62 | 18 |  | 1.94 (0.81-4.68) | 0.1 |  |  | 1.85 (0.74-4.62) | | 0.2 |  |  | 1.45 (0.54-3.88) | 0.5 |  |
| Q1 (≤20) | 70 | 25 |  | 3.00 (1.28-7.04) | 0.01 |  |  | 2.46 (0.98-6.21) | | 0.06 |  |  | 1.57 (0.57-4.35) | 0.4 |  |
| Overprotection |  |  |  |  |  |  |  |  | |  |  |  |  |  |  |
| Q1 (≤4) | 69 | 8 |  | 1.00 |  | 0.005 |  | 1.00 | |  | 0.03 |  | 1.00 |  | 0.2 |
| Q2 (5-9) | 73 | 21 |  | 2.93 (1.17-7.34) | 0.02 |  |  | 2.73 (1.05-7.14) | | 0.0402 |  |  | 2.43 (0.88-6.72) | 0.09 |  |
| Q3 (10-13) | 53 | 12 |  | 2.45 (0.89-6.75) | 0.08 |  |  | 1.98 (0.69-5.70) | | 0.2 |  |  | 1.79 (0.59-5.49) | 0.3 |  |
| Q4 (≥14) | 70 | 25 |  | 4.27 (1.70-10.74) | 0.002 |  |  | 3.58 (1.36-9.45) | | 0.01 |  |  | 2.64 (0.93-7.54) | 0.07 |  |
| Mother |  |  |  |  |  |  |  |  | |  |  |  |  |  |  |
| Care |  |  |  |  |  |  |  |  | |  |  |  |  |  |  |
| Q4 (≥35) | 58 | 10 |  | 1.00 |  | 0.3 |  | 1.00 | |  | 0.4 |  | 1.00 |  | 0.7 |
| Q3 (29-34) | 80 | 21 |  | 1.48 (0.61-3.58) | 0.4 |  |  | 1.32 (0.52-3.35) | | 0.6 |  |  | 0.88 (0.32-2.37) | 0.8 |  |
| Q2 (25-28) | 57 | 17 |  | 1.91 (0.75-4.85) | 0.2 |  |  | 2.57 (0.96-6.86) | | 0.06 |  |  | 2.16 (0.78-6.03) | 0.1 |  |
| Q1 (≤24) | 70 | 18 |  | 1.62 (0.65-4.05) | 0.3 |  |  | 1.29 (0.49-3.42) | | 0.6 |  |  | 0.57 (0.19-1.72) | 0.3 |  |
| Overprotection |  |  |  |  |  |  |  |  | |  |  |  |  |  |  |
| Q1 (≤4) | 70 | 10 |  | 1.00 |  | 0.02 |  | 1.00 | |  | 0.05 |  | 1.00 |  | 0.3 |
| Q2 (5-8) | 52 | 15 |  | 2.55 (1.01-6.46) | <0.05 |  |  | 2.51 (0.94-6.72) | | 0.07 |  |  | 2.53 (0.88-7.30) | 0.09 |  |
| Q3 (9-13) | 81 | 21 |  | 2.54 (1.05-6.13) | 0.04 |  |  | 2.38 (0.94-6.02) | | 0.07 |  |  | 2.13 (0.80-5.68) | 0.1 |  |
| Q4 (≥26) | 62 | 20 |  | 3.10 (1.25-7.66) | 0.01 |  |  | 2.77 (1.04-7.33) | | 0.04 |  |  | 1.88 (0.64-5.51) | 0.3 |  |
| ***Women (n=437)*** |  |  |  |  |  |  |  |  | |  |  |  |  |  |  |
| Father |  |  |  |  |  |  |  |  | |  |  |  |  |  |  |
| Care |  |  |  |  |  |  |  |  | |  |  |  |  |  |  |
| Q4 (≥35) | 99 | 23 |  | 1.00 |  | 0.3 |  | 1.00 | |  | 0.7 |  | 1.00 |  | 0.3 |
| Q3 (30-34) | 124 | 44 |  | 1.86 (1.02-3.42) | 0.04 |  |  | 1.70 (0.91-3.19) | | 0.1 |  |  | 1.70 (0.88-3.26) | 0.1 |  |
| Q2 (23-29) | 108 | 35 |  | 1.52 (0.81-2.86) | 0.2 |  |  | 1.39 (0.72-2.67) | | 0.3 |  |  | 1.06 (0.53-2.12) | 0.9 |  |
| Q1 (≤22) | 106 | 35 |  | 1.62 (0.85-3.09) | 0.1 |  |  | 1.31 (0.67-2.56) | | 0.4 |  |  | 0.86 (0.41-1.77) | 0.7 |  |
| Overprotection |  |  |  |  |  |  |  |  | |  |  |  |  |  |  |
| Q1 (≤3) | 112 | 31 |  | 1.00 |  | 0.2 |  | 1.00 | |  | 0.4 |  | 1.00 |  | 0.7 |
| Q2 (4-7) | 106 | 31 |  | 1.08 (0.59-1.97) | 0.8 |  |  | 0.95 (0.51-1.77) | | 0.9 |  |  | 0.85 (0.44-1.63) | 0.6 |  |
| Q3 (8-12) | 109 | 33 |  | 1.03 (0.56-1.88) | 0.9 |  |  | 0.88 (0.47-1.65) | | 0.7 |  |  | 0.80 (0.41-1.54) | 0.5 |  |
| Q4 (≥33) | 110 | 42 |  | 1.58 (0.88-2.84) | 0.1 |  |  | 1.36 (0.74-2.50) | | 0.3 |  |  | 0.87 (0.45-1.68) | 0.7 |  |
| Mother |  |  |  |  |  |  |  |  | |  |  |  |  |  |  |
| Care |  |  |  |  |  |  |  |  | |  |  |  |  |  |  |
| Q4 (≥36) | 107 | 27 |  | 1.00 |  | 0.05 |  | 1.00 | |  | 0.1 |  | 1.00 |  | 0.9 |
| Q3 (32-35) | 125 | 36 |  | 1.20 (0.66-2.17) | 0.6 |  |  | 1.09 (0.58-2.02) | | 0.8 |  |  | 1.02 (0.53-1.96) | 0.9 |  |
| Q2 (27-31) | 94 | 30 |  | 1.36 (0.72-2.54) | 0.3 |  |  | 1.20 (0.63-2.28) | | 0.6 |  |  | 1.03 (0.53-2.02) | 0.9 |  |
| Q1 (≤26) | 111 | 44 |  | 1.79 (0.98-3.29) | 0.06 |  |  | 1.58 (0.84-2.96) | | 0.2 |  |  | 0.96 (0.48-1.89) | 0.9 |  |
| Overprotection |  |  |  |  |  |  |  |  | |  |  |  |  |  |  |
| Q1 (≤3) | 120 | 30 |  | 1.00 |  | 0.01 |  | 1.00 | |  | 0.04 |  | 1.00 |  | 0.5 |
| Q2 (4-7) | 107 | 30 |  | 1.15 (0.63-2.11) | 0.6 |  |  | 1.00 (0.54-1.88) | | 1 |  |  | 0.92 (0.48-1.77) | 0.8 |  |
| Q3 (8-12) | 101 | 34 |  | 1.60 (0.87-2.94) | 0.1 |  |  | 1.57 (0.84-2.92) | | 0.2 |  |  | 1.31 (0.68-2.50) | 0.4 |  |
| Q4 (≥33) | 109 | 43 |  | 1.99 (1.11-3.58) | 0.02 |  |  | 1.68 (0.91-3.08) | | 0.1 |  |  | 1.16 (0.61-2.23) | 0.6 |  |
| Model 1: Adjusted for sociodemographic and lifestyle factors (age, [sex for the first table], marital status, educational level, subjective economic level, occupation, current drinking, current smoking and habitual exercise.)  Model 2: Adjusted for Model 1 + physical factors (Obesity, hypertension, diabetes, past history of cardiovascular disease, past history of cancer, past history of respiratory diseases, past history of digestive diseases and current pain symptom.)  Model 3: Adjusted for Model 2 + depressive symptom as a psychological factor. | | | | | | | | | | | | | | | |
