# Supplementary material for: Perceived inadequate care and excessive overprotection during childhood are associated with greater risk of sleep disturbance in adulthood: the Hisayama Study
Source: BMC Psychiatry. 2016 Jul 7;16:215. doi: 10.1186/s12888-016-0926-2 (PMC4936292; doi:10.1186/s12888-016-0926-2)
Supplement: Additional file 3: Table S3. — Characteristics of participants and non-participants. (DOCX 15 kb) [file 12888_2016_926_MOESM3_ESM.docx]

| **Table S3. Characteristics of participants and non-participants.** | | |  |
| --- | --- | --- | --- |
|  | Participants | Others | P value |
|  | (n=702) | (n=1548) |  |
| ***Sociodemographic and lifestyle factors*** |  |  |  |
| Age, mean (SD) | 59.3 (11.0) | 66.0 (11.3) | <0.01 |
| Sex, male (%) | 37.8 | 45.3 | <0.01 |
| Marital status, without partner (%) | 18.2 | 21.8 | 0.1 |
| Occupation, unemployed (%) | 49.9 | 60.1 | <0.01 |
| Current smoking, yes (%) | 10.8 | 14.9 | <0.01 |
| Current drinking, yes (%) | 54.4 | 47.2 | <0.01 |
| Habitual exercise, yes (%) | 52.9 | 52.6 | 0.9 |
| ***Physical factors*** |  |  |  |
| Obesity, BMI ≥ 25 (%) | 23.9 | 26.2 | 0.3 |
| Hypertension (%) | 41.7 | 57.6 | <0.01 |
| Diabetes (%) | 15.0 | 17.4 | 0.2 |
| Past history of cardiovascular diseases (%) | 13.5 | 17.0 | 0.04 |
| Past history of cancer (%) | 6.1 | 9.5 | <0.01 |
| Past history of respiratory diseases (%) | 14.7 | 16.0 | 0.4 |
| Past history of digestive diseases (%) | 20.8 | 25.6 | 0.01 |
| Values are expressed as mean (standard deviation [SD]) or frequency.  Hypertension was defined as blood pressure ≥ 140/90 mm Hg and/or use of an antihypertensive agent. Diabetes was defined as a fasting plasma glucose level ≥ 7.0 mmol/L (126 mg/dL) and/or a 2-h post-loaded or causal glucose level ≥ 11.1 mmol/L (200 mg/dL), HbA1c (NGSP) ≥ 6.5% and/or current use of insulin or oral glucose-lowering agents. Values were tested by t-test (for age) or Chi-square test (for frequencies). | | | |
